# Study on Immunoregulatory Effects of Fucoidan from *Sargassum graminifolium* In Vivo and Immunoactivation Activity of Its Fecal Fermentation Products Using Co-Culture Model

**DOI:** 10.3390/molecules28237794

**Published:** 2023-11-27

**Authors:** Cuifang Wang, Lan Huang, Yaolong Huang, Xin Tian, Jieqing Liu

**Affiliations:** 1 College of Oceanology and Food Science, Quanzhou Normal University, Quanzhou 362000, China; huangyaolong@stumail.qztc.edu.cn (Y.H.); 201005017@stumail.qztc.edu.cn (X.T.); 2 Fujian Province Key Laboratory for the Development of Bioactive Material from Marine Algae, Quanzhou Normal University, Quanzhou 362000, China; 3 School of Medicine, Huaqiao University, Quanzhou 362021, China; hl287216411@163.com

**Keywords:** *Sargassum graminifolium*, fucoidan, immunomodulatory effect, fecal fermentation, Caco-2/RAW264.7 co-culture

## Abstract

Fucoidan, brown seaweed-derived dietary fibers (DFs), can be considered a promising candidate for modulating immune responses. Due to its structural complexity and diversity, it is unclear whether *Sargassum graminifolium* fucoidans (SGFs) also show marvelous immunoregulatory effects. In the present study, two fractions, SGF−1 and SGF−2, were purified from SGFs by DEAE-Sepharose Fast Flow and Sephacryl S-400 HR column chromatography. We investigated the in vivo immune regulatory activity of SGF−2 and explored the immune activation of SGF−2 fecal fermentation products with in vitro fecal fermentation combined with a Caco-2/RAW264.7 co-culture system. In vivo results exhibited that SGF−2 could elevate the thymus/spleen indices, CD8^+^ splenic T lymphocyte subpopulations, and CD4^+^ Foxp3^+^ splenic Tregs. The 16S high-throughput sequencing results showed that SGF−2 administration significantly increased the relative abundance of *Lactobacillus*, *Alloprevotella*, *Ruminococcus*, and *Akkermansia*. In addition, it was found that SGF−2 fermented by feces could significantly improve the phagocytosis, NO, and cytokine (TNF−α, IL−6, and IL−10) production of macrophages in the co-culture system. These results indicated that SGFs have the potential to modulate immunity and promote health by affecting the gut microbiota.

## 1. Introduction

Dietary fiber (DF) is a class of edible carbohydrate polymers that are not digested and absorbed by the body but can be metabolized by gut microorganisms into beneficial metabolites, including short-chain fatty acids (SCFAs) [1]. DFs and SCFAs can contribute to host health by preventing dysbiosis of gut microbial ecology and maintaining metabolic homeostasis [2]. Brown seaweeds (Phaeophyta) have been a source of food in many countries since ancient times. Currently, brown seaweed-derived polysaccharides including alginate, fucoidan, and laminarins have been considered DFs with a potential prebiotic effect [3]. Fucoidan, which is rich in fucose and sulfuric acid groups, is mainly distributed in the cell walls of brown seaweeds. Because of its complex chemical structure, large molecular weight, and multi-branch structure, it is difficult to determine the fucoidan structure. The current studies indicate that it mainly possesses two predominant types of backbones, including a repeating (1 → 3) linked *α*-_L_-fucopyranose (type I) and an alternating (1 → 3)- and (1 → 4)-linked *α*-_L_-fucopyranose (type II) [4].

*Sargassum graminifolium*, belonging to the genus *Sargassum*, is an edible marine brown alga widely distributed along the southeast coast of China. Previously, there were few reports to evaluate the polysaccharide bioactivity of *S. graminifolium.* These few studies indicated that crude polysaccharides from *S. graminifolium* exhibited antioxidant properties and a protective effect on ethylene glycol-induced kidney damage [5,6]. Our recent studies indicated that fucoidan and alginate from *S. graminifolium* might relieve the symptoms of food allergy in OVA-induced mice by regulating gut microbiota [7]. However, the immunomodulatory effect of polysaccharides from *S. graminifolium* and its underlying mechanisms need to be investigated. In addition, the previous studies mainly evaluated the biological activity of crude polysaccharides from *S. graminifolium* without evaluating the biological function of their purification fractions. Therefore, this study aimed to further isolate and purify crude polysaccharides and evaluate the immunomodulatory activity of its purified fractions in vivo.

In recent years, an in vitro fecal fermentation model has been widely established to effectively simulate the process of decomposing and utilizing polysaccharides by intestinal microorganisms [8,9]. This method has the unique advantages of good reproducibility, effective control, and simplicity, but also has the obvious disadvantage that it can only study the polysaccharides’ metabolism and their effects on microorganisms, without involving intestinal epithelial cells (IECs) and immune cells. Moreover, a novel in vitro co-culture model consisting of Caco-2 cells (in the upper layer of a Transwell) and immune cells (in the lower layer of the Transwell) was constructed to evaluate the sensitization potential of foods [10]. Hence, an in vitro fecal fermentation model combined with a co-culture system can effectively simulate the intestinal microenvironment and explore the interaction between polysaccharides and the intestine, providing the possibility for further exploration of the immunomodulatory mechanism of fucoidan. In this study, in vitro fecal fermentation combined with a Caco-2/RAW264.7 cell co-culture model was utilized to explore the immune-activating effect of SGFs with fecal fermentation through IECs.

## 2. Results and Discussion

### 2.1. Purification and Characterization of SGF−1 and SGF−2

**Figure 1:**
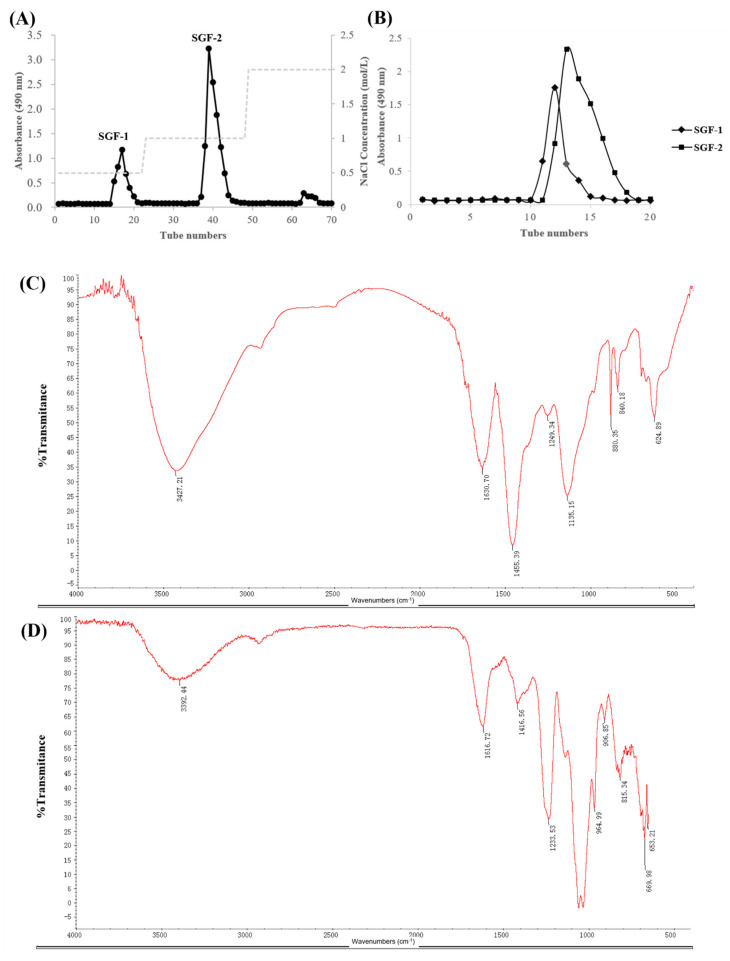
Purification and characterization of SGF-−1 and SGF−2. (**A**) Elution pattern of the purified fucoidan by DEAE-Sepharose Fast Flow chromatography. (**B**) Elution pattern of SGF−1 and SGF−2 by Sephacryl S−400 HR column. (**C**,**D**) FTIR spectrum of SGF−1 and SGF−2.

**Figure 2:**
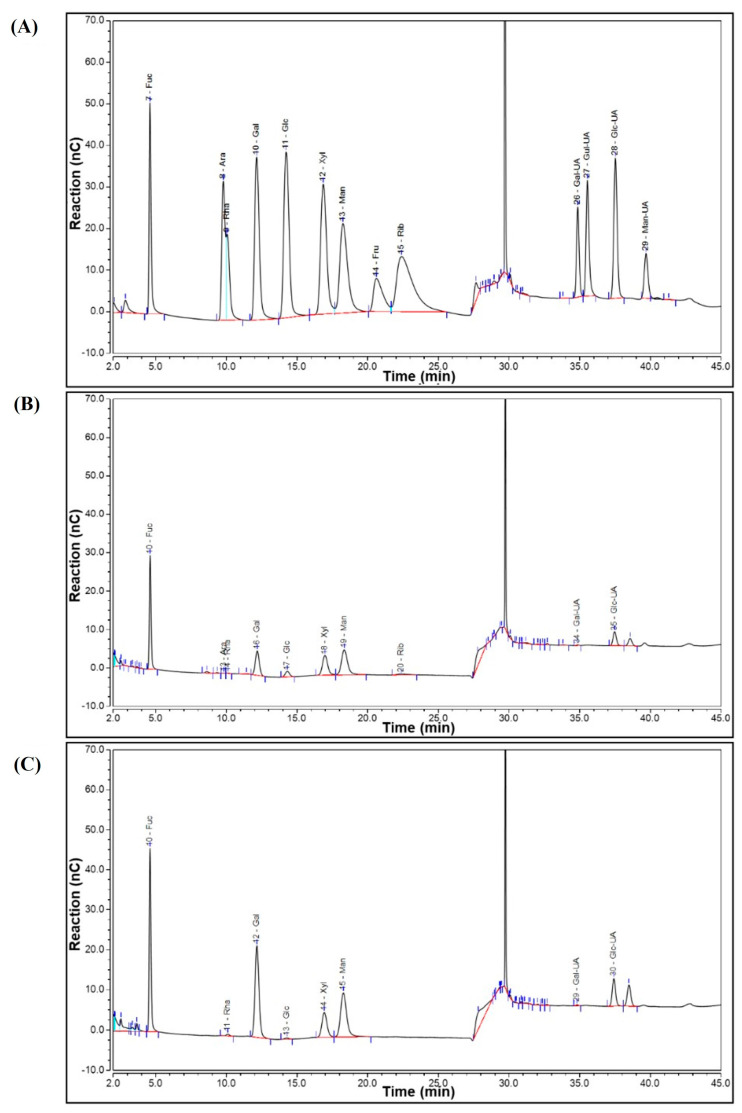
Ion chromatogram of monosaccharides SGF−1 and SGF−2. (**A**) standard ion chromatogram; (**B**) ion chromatogram of SGF−1; (**C**). ion chromatogram of SGF−2. The black curve is the absorption curve. The red curve represents the line connecting the starting and ending points of the integrated area.

The crude fucoidan extracted from *S. graminifolium* was further purified by DEAE-Sepharose Fast Flow chromatography. As shown in Figure 1A,B, two main fractions, A (eluted with 0.5 mol/L NaCl) and B (eluted with 1 mol/L NaCl), were collected, desalted, and then purified by a Sephacryl S-400 HR column to obtain SGF−1 and SGF−2. The analysis of physicochemical properties is shown in Table 1: SGF−1 is a fucoidan with 74.72% total sugar content, 5.00% sulfate content, and an average molecular weight of 112.96 kDa, and SGF−2 is a fucoidan with 55.79% total sugar content, 31.81% sulfate content, and an average molecular weight of 258.07 kDa. Further analysis of the monosaccharide composition revealed that both SGF−1 and SGF−2 are mainly composed of Fuc, Man, Gal, Xyl, and Glc-UA, with Fuc being the main constituent in the structure of SGF−1 and SGF−2, accounting for 40.54% and 35.57%, respectively (Table 1 and Figure 2). The functional groups and chemical bond analysis of SGF−1 and SGF−2 were determined by Fourier transform infrared (FTIR) spectroscopy (Figure 1C,D). The results indicated that there are some similar functional groups in the structure of SGF−1 and SGF−2, including O-H stretching vibration (a broad stretching peak at 3300–3600 cm^−1^), C-H stretching vibration (a mall stretching peak near 2800 cm^−1^), and C=O asymmetric stretching vibration (a stretching peak near 1620 cm^−1^) [11]; however, significantly different stretching vibrations between SGF−1 and SGF−2 can be observed in the absorption peak around 1230 cm^−1^. An obvious 1224 cm^−1^ absorption peak, which was assigned to the sulfate group, was present in SGF−2, while a small sulfate group peak at 1249 cm^−1^ was observed in SGF−1, which is consistent with the previous results of high sulfate content in SGF−2 (31.81%) and low sulfate content in SGF−1 (5.00%). Although two fractions, SGF−1 and SGF−2, were purified from the fucoidan of *S. graminifolium*, only the immunomodulatory activity of fraction SGF−2 was further explored in the following experiments due to the low yield found in SGF−1.

### 2.2. The Effects of SGF−2 on Immune Organ Indices

The effects of SGF−2 on body, spleen, and thymus weights are presented in Table 2. The Con group refers to mice who were administered with sterile physiological saline, the FL group refers to mice administered with a low dose of SGF−2 (125 mg/kg body weight), and the FH group refers to mice administered with a high dose of SGF−2 (250 mg/kg body weight). The results showed that there is no significant difference in weight gain among the Con group (29.93%), the FL group (30.72%), and the FH group (29.90%). The spleen and thymus indices of the mice in the Con group were 4.30 ± 0.98 mg/g and 1.63 ± 0.62 mg/g, respectively, while in the FH group, the spleen and thymus indices of the mice significantly increased to 5.34 ± 0.58 mg/g and 2.07 ± 0.75 mg/g, respectively (*p* < 0.05).

### 2.3. Modulation of Gut Microbiota by SGF−2

**Figure 3:**
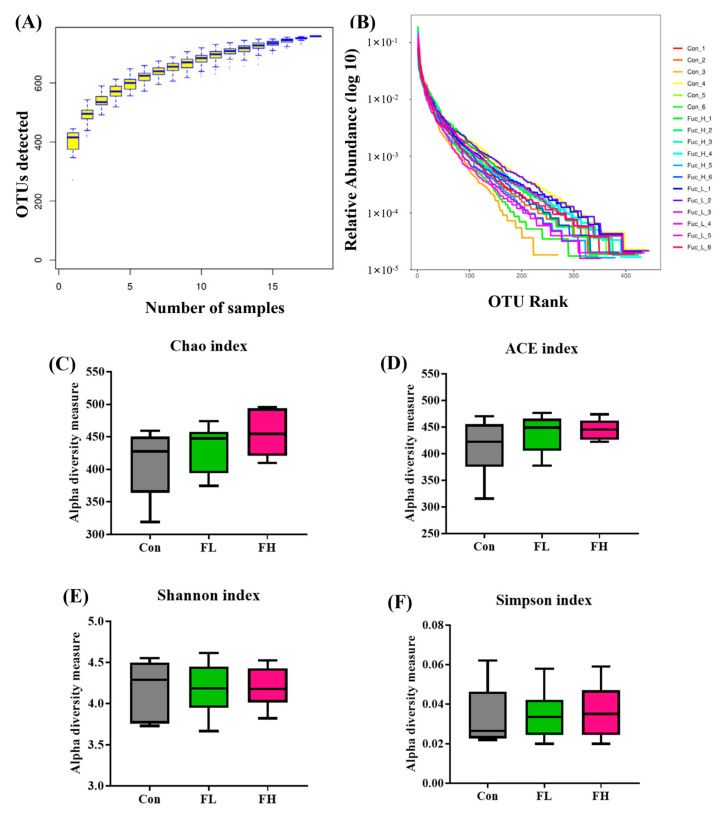
Effect of SGF−2 on the diversity of gut microbiota in mice (*n* = 6). (**A**) Species accumulation curve. (**B**) Rank abundance curve. (**C**) Chao index. (**D**) ACE index. (**E**) Shannon index. (**F**) Simpson index. Con, mice gavaged at the same volume of sterile physiological saline. FL, mice gavaged at low dose SGF−2 (125 mg/kg body weight). FH, mice gavaged at high dose SGF−2 (250 mg/kg body weight). *p* > 0.05 vs. Con group.

Accumulating data have demonstrated that human intestinal flora is important in modulating the nutrition, metabolism, and immunity of its host [12,13]. Following four weeks of treatment, six mice per group were randomly selected to have their intestinal contents collected. These intestinal contents underwent DNA extraction and high-throughput sequencing to check whether SGF−2 could exert a beneficial role by regulating the gut microbiota. After selecting the effective reads, the dataset consisted of 423,596 reads from the Con group (*n* = 6, with 70,599 ± 2851 reads/sample), 420,325 reads from the FL group (*n* = 6, with 70,054 ± 1945 reads/sample), and 421,350 reads from the FH group (*n* = 6, with 70,225 ± 3415 reads/sample). The species accumulation curve can be used to estimate the adequacy of the sample quantity and determine the species richness. A rank abundance curve is an effective tool to investigate species evenness and abundance. If the curve is smoother, the species composition of the sample will be more uniform. As shown in Figure 3A,B, the species accumulation curves showed a sharp rise and then maintained stability, and the rank abundance curves are smooth, indicating that these data can effectively reflect the uniformity and richness of the gut microbiota. Next, an α-diversity analysis of each sample was performed to assess the species richness (Chao and Ace) and species diversity (Simpson and Shannon). However, interestingly, there was no significant change in intestinal community richness and diversity after the fucoidan intervention, compared to the control group (Figure 3C–F).

**Figure 4:**
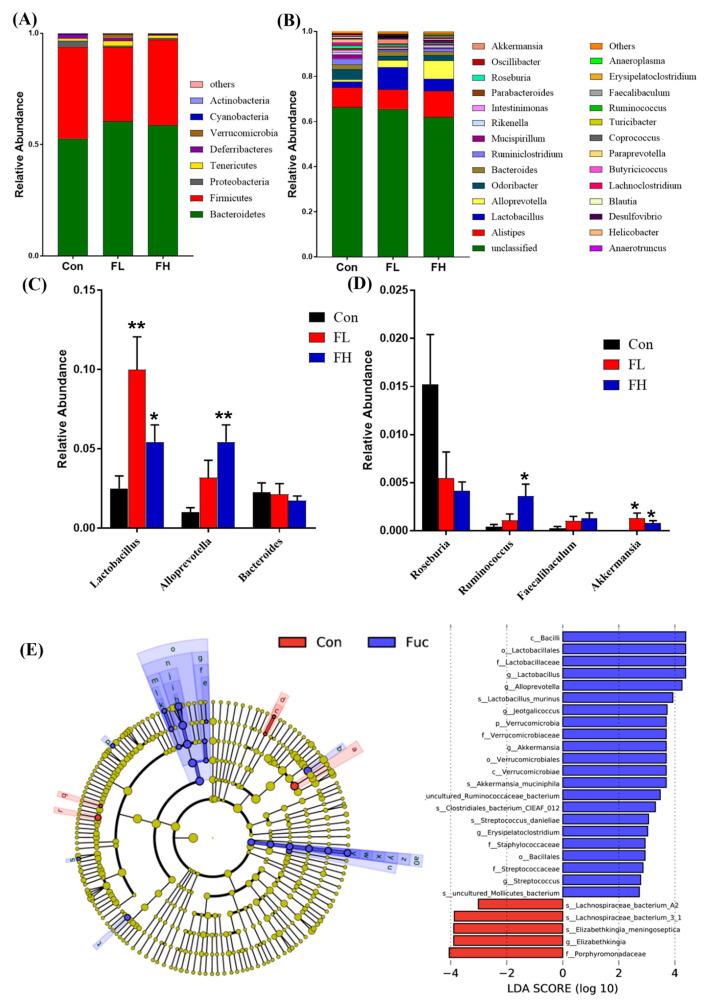
Effect of SGF−2 on the composition of gut microbiota in mice (*n* = 6). (**A**) The intestinal flora at the phylum level. (**B**) The intestinal flora at the genus level. (**C**) The relative abundance of *Lactobacillus*, *Alloprevotella*, and *Bacteroides*. (**D**) The relative abundance of *Roseburia*, *Ruminococcus*, *Faecalibaculum*, and *Akkermansia*. (**E**) LEfSe analysis. Con, mice gavaged at the same volume of sterile physiological saline. FL, mice gavaged at low dose SGF−2 (125 mg/kg body weight). FH, mice gavaged at high dose SGF−2 (250 mg/kg body weight). * *p* < 0.05, ** *p* < 0.01 vs. Con group.

To better understand the structural response of the intestinal flora before and after SGF−2 administration, the taxonomic compositions of the three groups were then compared. At the phylum level, the relative abundance of the three groups is shown in Figure 4A. The *Bacteroidetes* and *Firmicutes* in the control group were the main dominant bacterial communities, accounting for 55.90% and 36.47%, respectively. In the FL and FH groups, the intestinal microflora was still dominated by *Bacteroidetes* and *Firmicutes; Bacteroidetes* increased slightly, whereas *Firmicutes* showed a mild decrease after being treated with SGF−2, but there was no significant difference. It has been reported that *Firmicutes* and *Bacteroidetes* contain many glycoside hydrolases that can degrade non-digestible polysaccharides [14]. In addition, more carbohydrate metabolic pathways and glycoside hydrolases were found in *Bacteroidetes* compared to *Firmicutes;* these pathways and glycoside hydrolases play an important role in fermenting glycans into SCFAs [15]. At the genus level, *Alistipes*, *Lactobacillus*, *Alloprevotella*, *Odoribacter*, *Bacteroides*, and *Ruminiclostridium* were the dominant bacteria (Figure 4B). Compared to the control group, the relative abundance of *Lactobacillus* significantly increased in the FL group (*p* < 0.01) and FH group (*p* < 0.05), and the relative abundance of *Alloprevotella* also significantly increased in the FH group (*p* < 0.01) (Figure 3C). Moreover, the genera with low relative abundance, including *Roseburia*, *Ruminococcus*, *Faecalibaculum*, and *Akkermansia*, were also found to be contained in different proportions in the three groups (Figure 4D). After the SGF−2 treatment, the relative abundance of *Roseburia* was decreased, while *Ruminococcus*, *Faecalibaculum*, and *Akkermansia* were increased. It has been reported that *Lactobacillus*, *Ruminococcus*, and *Faecalibaculum*, belonging to *Firmicutes*, and *Alloprevotella*, belonging to *Bacteroidetes*, are short-chain fatty acid (SCFA)-producers [16]. SCFAs (acetate, propionate, and butyrate), the final product of intestinal microbial fermentation of dietary fibers and other undigested carbohydrates, play an important role in immune system regulation [17]. Regulatory T cells (Tregs) are critical for maintaining immune reactivity to self-antigens and for inhibiting excessive immune responses harmful to the host [18]. SCFAs could promote the differentiation of T cells into Tregs to promote immunity or immune tolerance [19]. These results suggest that SGF−2 could regulate immunity, possibly by increasing the number of probiotics, which can produce SCFAs. Additionally, it is worth noting that the SGF−2 treatment also increased the abundance of *Akkermansia*, whose abundance was negatively correlated with obesity, diabetes, and cardiovascular diseases [20,21]. Recently, *Akkermansia* has also been illustrated to restore the percentage of Tregs [22], indicating the potential of *Akkermansia* to regulate immunity. As such, in both the FL and FH groups, the treatment significantly increased the relative abundance of *Akkermansia*, so it is reasonable to attribute the benefits of SGF−2 in part to its regulation of this gut microbe.

The dominant bacterial taxa in the control group and SGF−2 treatment groups were further identified using the linear discriminant analysis effect size (LEfSe) method, and the results are shown in Figure 4E. The microbial community in the control group and SGF−2 treated groups were completely different. Consistent with the above results, the SGF−2 treated groups were enriched in *Lactobacillus*, *Alloprevotella*, and *Akkermansia* at the genus level, while the control group was enriched in *Lachnospiraceae* and *Elizabethkingia*.

### 2.4. Regulatory Effect of SGF−2 on Immune Cell Subpopulations

**Figure 5:**
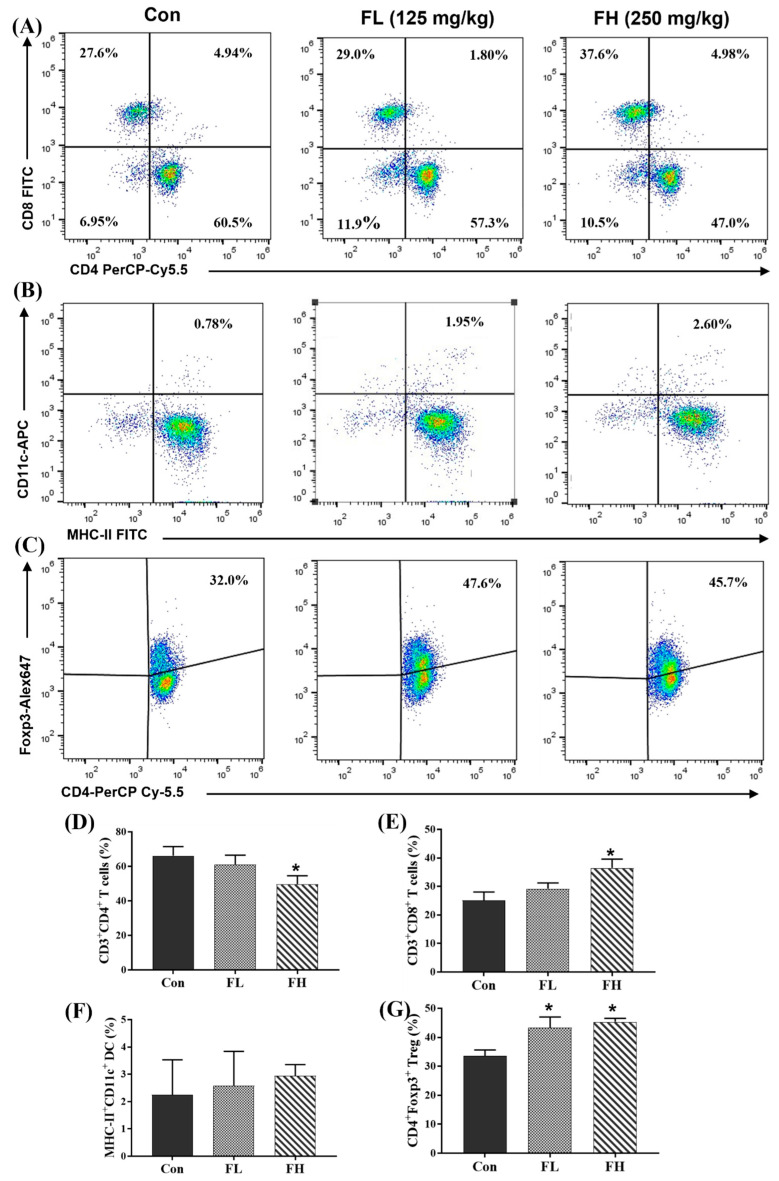
Flow cytometry analyses of subsets of T lymphocytes, Tregs, and DCs (*n* = 3). (**A**). Effect of SGF−2 on T lymphocytes. (**B**). Effect of SGF−2 on DCs. (**C**). Effect of SGF−2 on Tregs. (**D**–**G**). The statistical analysis of cell subpopulations. Con, mice gavaged at the same volume of sterile physiological saline. FL, mice gavaged at a low dose of SGF−2 (125 mg/kg body weight). FH, mice gavaged at a high dose of SGF−2 (250 mg/kg body weight). * *p* < 0.05 vs. con group.

To investigate the effect of SGF−2 on immune cell subsets, mouse spleen lymphocytes were taken, and flow cytometry was used to analyze the numbers of T lymphocytes, DCs, and Tregs. As shown in Figure 5A,D,E, the subpopulation of CD8^+^ gated from CD3^+^ T cells was significantly increased after the SGF−2 treatment (FH group), while CD4^+^ gated from CD3^+^ T cells was significantly decreased after the SGF−2 treatment (FL and FH groups). The DCs in the mouse splenocytes were defined by CD11c^+^ MHC-II^+^, and the flow cytometry result showed that there were no significant differences in the percentage of CD11c^+^MHC-II^+^ DCs among the three groups (Figure 5B,F). Additionally, the CD4^+^Foxp3^+^ Treg subsets in the mouse splenocytes were also analyzed. Compared to the normal mouse splenocytes (32.0%), the percentage of CD4^+^Foxp3^+^ Tregs in the splenocytes of the SGF−2-treated mice (FL and FH) significantly increased to 47.6% and 45.7%, respectively (Figure 5C,G).

The immunomodulatory effects of polysaccharides can be achieved by activating effector cells including macrophages, lymphocytes, and dendritic cells [23]. Fucoidans from *Fucus vesiculosus* and *Undaria pinnatifida* have been found to activate natural killer (NK) cells and T cells, promote DC maturation, and then promote pro-inflammatory cytokine production [24,25]. Our results indicated that SGF−2 elevated the number of CD3^+^CD8^+^ T cells and CD4^+^ Foxp3^+^ Tregs, which to some extent verified the immunostimulatory activity of SGF−2. The critical roles of CD4^+^Foxp3^+^ Tregs in immune regulation and the fact that SCFAs can promote the differentiation of T cells into Tregs have been discussed above. Here, we demonstrated that SGF−2 treatment could promote the production of Tregs, suggesting that fucoidans induce Treg differentiation by increasing the production of SCFAs by gut microbiota. However, the conclusion that SGF−2 treatment can increase SCFA levels still needs to be verified by detecting the levels of SCFAs in mouse serum. In addition, SCFAs can enhance the production of IL-10 by DCs through G-coupled receptors to promote the differentiation of Tregs [26]. However, in our results, no obvious changes in the number of DCs (CD11c^+^MHC-II^+^) were observed after SGF−2 treatment.

### 2.5. Effect of SGF−2 with Fecal Fermentation on the Immunoregulatory Activity in a Co-Culture Model

**Figure 6:**
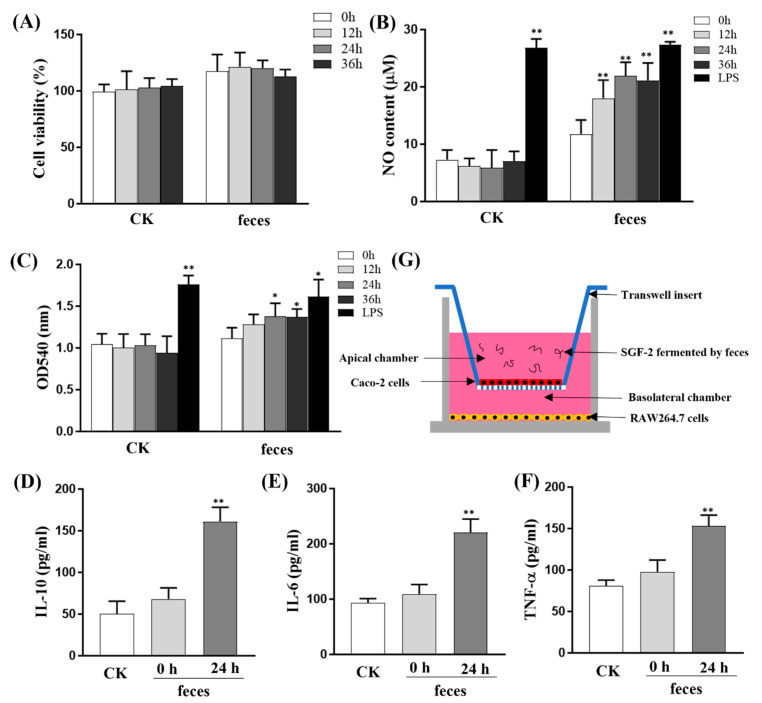
Activation effects of SGF−2 fermented by feces in co-culture model (*n* = 3). (**A**) Cell viability. (**B**) NO production. (**C**) Phagocytic activity. (**D**–**F**) IL-10, IL-6, and TNF-α levels. (**G**) The Caco-2/RAW264.7 co-culture system. CK, fermentation flasks with sterile physiological saline. Feces, fermentation flasks with SGF−2. * *p* < 0.05, ** *p* < 0.01 vs. CK group.

In recent years, a series of connections have emerged between host physiology, the composition of the gut microbiota, and DFs. The main view is that the combination of plant fiber with fiber-digesting probiotics could increase the components of immune regulation activity and further modulate the host physiology by transmitting signals to immune cells through pattern recognition receptors (PRRs) of IECs [26]. Therefore, an in vitro fecal fermentation model combined with a Caco-2/RAW264.7 co-culture system was established to simulate the gut microenvironment and explore the mechanisms of SGF−2 immune regulation (Figure 6G).

#### 2.5.1. Effects of SGF−2 with Fecal Fermentation on Cell Viability in RAW264.7 Cells

After being treated with SGF−2 fecal fermentation products for 24 h, the CCK8 assay was used to examine the cytotoxic effects of RAW264.7 macrophages in the basal chamber. As shown in Figure 6A, there were no significant changes in cell viability of RAW264.7 macrophages between the control group (CK) and SGF−2 fecal fermentation supernatant treatment group (feces). Moreover, there was no obvious change in cell viability upon variation of fermentation time (i.e., 0, 12, 24, and 36 h).

#### 2.5.2. Effects of SGF−2 with Fecal Fermentation on Phagocytic Activity

Phagocytic activity is a main indicator of the functional activation of macrophages. Hence, the phagocytic activity of RAW264.7 macrophages in the basal chamber was determined after being treated with SGF−2 fecal fermentation products for 24 h. As shown in Figure 6C, no obvious change in phagocytic activity of RAW macrophages in the co-culture system was observed between the control group (CK) and SGF−2 fecal fermentation supernatant treatment group (fermentation time: 0 h and 12 h), while with the increase in SGF−2 fermentation time (24 h and 36 h), the phagocytic activities were significantly enhanced after treatment by SGF−2 fermentation products (*p* < 0.05). Considering that the addition of SGF−2 fermented by feces in the co-culture system did not significantly affect the macrophage cell viability, it was eventually revealed that SGF−2 fermented by feces may partially enhance the phagocytic activity of RAW264.7 cells in the basal chamber.

#### 2.5.3. Effects of SGF−2 with Fecal Fermentation on the Levels of NO and Cytokines

Phagocyte nitric oxide (NO), which is the hallmark of macrophage activation, can effectively prevent the invasion of pathogens. Hence, the detection of NO content in the culture medium can be used as a reliable method to evaluate the activation of macrophages [27]. To evaluate the potential immunostimulatory activity of SGF−2 fermented by feces in the co-culture system, Griess reagent was used to measure the NO content in the basal culture medium (Figure 6B). The results showed that the NO content in the culture medium of the basal chamber did not significantly increase after being treated with SGF−2 fecal fermentation products (fermentation time: 0 h). This result was inconsistent with the previous reports that fucoidan from *Nizamuddinia zanardinii* could stimulate macrophages to secrete NO [28]. The reason may be that the fucoidan was not fermented by feces in this group, and the final concentration (1 μg/mL) is much lower than that in the literature (10 μg/mL). With the extension of the fermentation time of the SGF−2, the levels of NO secreted by macrophages gradually increased, and the SGF−2 with fecal fermentation (fermentation time: 24 h) showed the most significant impact on the NO production in the co-culture system. These results showed that SGF−2 fermented by feces can promote macrophage stimulation in the co-culture model.

Based on the above results, the cytokine levels (TNF-α, IL-6, and IL-10) in the co-culture model were detected by specific ELISA experiments (CK group, SGF−2 with fecal fermentation for 0 h, and SGF−2 with fecal fermentation for 24 h). As shown in Figure 6E,F, the proinflammatory cytokine levels, including IL-6 and TNF-α, did not significantly increase after being treated with SGF−2 fermentation products (fermentation time: 0 h), while the IL-6 and TNF-α levels significantly increased upon treatment with SGF−2 fermentation products (fermentation time: 24 h). The IL-10 levels showed similar results (Figure 6F). Accordingly, the results of cytokine production of RAW264.7 macrophages including TNF-α, IL-6, and IL-10 showed a similar increasing trend to NO secretion in the co-culture system.

Macrophages play critical roles in immune regulation by producing inflammatory factors and pro-inflammatory cytokines. Previous studies have shown that fucoidan can stimulate macrophages to secrete NO, TNF-α, and IL-6 [28,29]. In this study, it was demonstrated that SGF−2 fermented by feces could significantly enhance macrophage activation by releasing IL-6, TNF-α, and NO in the Caco-2/RAW264.7 co-culture system. Some studies on in vitro fecal fermentation of undigested polysaccharides showed that SCFA concentration and the abundance of microbiota related to SCFA metabolism increased [8,30]. SCFAs can bind to SCFA receptors of IECs to regulate immune cells (DCs, T cells, and B cells) and are recognized as energy sources for IECs [31]. In this study, we demonstrated that SGF−2 fermented by feces can act on the Caco-2 monolayer to activate RAW264.7 macrophages, which are located on the basolateral side of the Transwell. In addition, SGF−2 fermented by feces can significantly increase IL-10 levels. Previous studies have shown that cytokine IL-10 can play a pivotal role in regulating the differentiation and proliferation of Foxp3^+^ Tregs [32]. This result is consistent with the above conclusion that SGF−2 up-regulates the subsets of Tregs in vivo.

In the present study, we included a detailed study on the isolation and purification of polysaccharides from *S. graminifolium* and then evaluated the immune activity of SGF−2 in vivo and in vitro. Up to now, the immune activity of purified fractions from *S. graminifolium* has been poorly documented. By including the activity evaluation of a purified fraction of SGF−2, it will be possible to interpret the data more soundly and hopefully to the reproducibility of studies. Our work adds an additional level of complexity in interpreting the mechanisms by which DFs contribute to host health. It is generally considered that DFs can be metabolized by gut microorganisms into beneficial metabolites including short-chain fatty acids (SCFAs) [1]. This conclusion is strengthened by our observation that SGF−2 fermented products enhanced immunoactivation activity in the Caco-2/RAW264.7 co-culture system. However, our work only discusses the effect of SGF−2 fermented products in the Caco-2/RAW264.7 co-culture system, which cannot fully reflect the real intestine environment. In recent studies, it has been demonstrated that SCFAs, the gut microbiota metabolites of DFs, can increase Foxp3+ Tregs by acting on IECs receptors GPR43 and GPR109a [26]. These cell surface receptors are not only expressed on IECs but also on intestinal immune cells, including DCs and Tregs. SCFAs could promote the production of Tregs by recognizing the GPR109a receptors on DCs [33]. In the following work, we can continue to establish the Caco-2/DCs co-culture system or Caco-2/T cell co-culture system to systematically explore the immunoactivation activity of SGF−2 fermented products. In addition, we only conducted a preliminary structure characterization of SGF−2, including FTIR analysis, molecular weight determination, and monosaccharide composition analysis. In future work, it will be necessary to determine its predominant types of bonding structure by 1D and 2D nuclear magnetic resonance (NMR) and by GC–mass spectrometry analysis.

## 3. Materials and Methods

### 3.1. Reagents and Materials

DEAE-Sepharose Fast Flow and Sephacryl S-400 HR were purchased from Shanghai Yuanye Bio-Technology Co., Ltd. (Shanghai, China). Detoxi-Gel endotoxin removing gel was obtained from Thermo Fisher Scientific (Waltham, MA, USA). Dimethyl sulfoxide (DMSO), lipopolysaccharide (LPS), vitamin K1, resazurin, and standard monosaccharides were acquired from Sigma-Aldrich (St. Louis, MO, USA). Yeast extract, peptone, hemin, L-cysteine, bile salts, NaHCH_3_, NaCl, KH_2_PO_4_, K_2_HPO_4_, CaCl_2_·6H_2_O, MgSO_4_·7H_2_O, and Tween-80 were obtained from Sinopharm Chemical Reagent Co., Ltd. (Ningbo, China). Methanol, ethyl acetate, acetone, and dichloromethane were acquired from Tianjing Chemical Reagents Co. (Tianjing, China).

Trypsin-EDTA, fetal bovine serum (FBS), and Dulbecco’s Modified Eagle’s medium (DMEM) were obtained from Gibico BRL (Grand Island, NY, USA). ELISA kits of IL-6, TNF-α, and IL-10 were acquired from R&D Systems (Minneapolis, MN, USA). The spleen lysis buffer kit was purchased from Sorlabio Co. (Beijing, China). Griess reagent, BCA, and CCK-8 kit were acquired from Beyotime Biotech (Shanghai, China). (FITC)-conjugated-CD8, APC-conjugated CD3, FITC-conjugated MHC-II, APC-conjugated CD11c, PerCP-Cy5.5-conjugated CD4, and Alexa Fluor 647 Foxp3 were acquired from Biolegend (San Diego, CA, USA).

### 3.2. Isolation of SGF−1 and SGF−2 from Sargassum graminifolium

**Figure 7:**
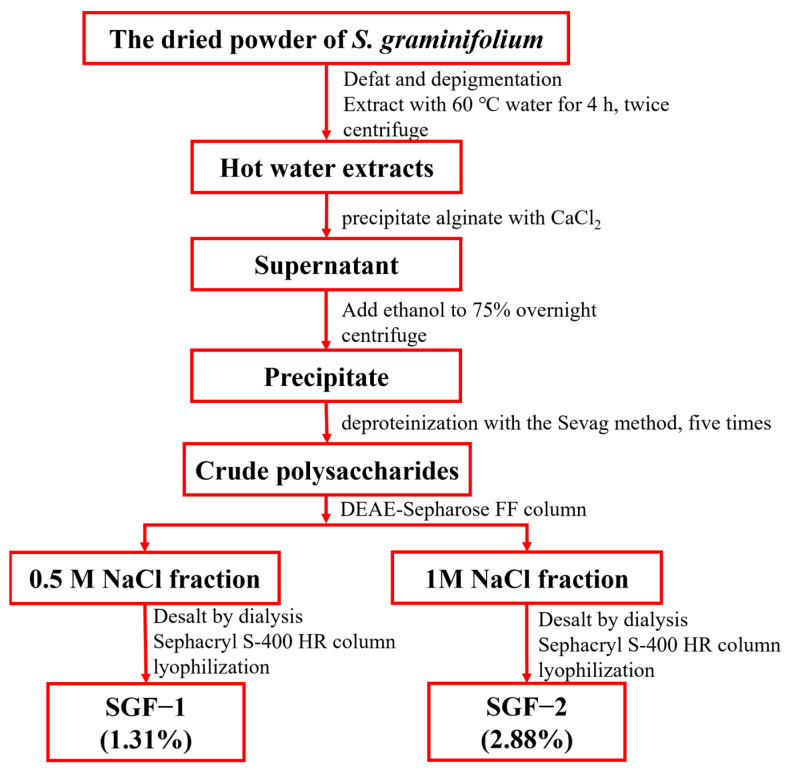
Stepwise extraction and purification procedure of SGF−1 and SGF−2 from *S. graminifolium.*

The crude fucoidans were prepared by referring to our previous studies [7], and the separation and purification process is shown in Figure 7. Briefly, the dried algae powder was extracted in water at 80 °C and precipitated with ethanol. The CaCl_2_ method was used to precipitate alginic acid. Thereafter, the polysaccharide was deproteinized five times by the Sevag method. Then, the crude fucoidan solution was purified and isolated by an anion exchange column (DEAE-Sepharose Fast Flow) with a gradient elution of 0.5 mol/L of NaCl and 1.0 mol/L of NaCl, respectively (10 mL/tube, flow rate: 5 mL/min). The eluents under the same elution peak were combined and desalinated using dialysis bags (1000 Da) according to the elution curve plotted by the phenol-sulfuric acid method [34]. A Sephacryl S-400 HR chromatograph was used to further purify the collected fucoidan fractions (eluted with ddH_2_O, flow rate: 1.5 mL/min, 3.0 mL/tube). Similarly, the eluents were pooled under the same elution peak. After lyophilization, two yellow powders were obtained, named SGF−1 and SGF−2. The contamination of endotoxin in the SGF−1 and SGF−2 was removed by Detoxi Endotoxin Removing Gel.

### 3.3. Physicochemical Properties Analysis of SGF−1 and SGF−2

The sugar content, sulfate content, and protein content of the SGF−1 and SGF−2 were determined by the phenol-sulfuric acid method, the BaCl_2_-gelatin method [35], and the BCA concentration determination kit [36], respectively. SEC-MALLS-RI was used to measure the average molecular weight of the SGF−1 and SGF−2 [37]. High-performance anion-exchange chromatography (HPAEC) was used to analyze the monosaccharide composition of the SGF−1 and SGF−2 [11]. A FTIR spectrometer (Vertex 70, Bruker, Mannheim, Germany) was used to detect the stretching/bending vibrations of the SGF−1 and SGF−2.

### 3.4. Animals and Experimental Design

All the following experimental procedures were approved by the relevant laws and conducted at the Experimental Animal Center of Huaqiao University of School of Medicine (approval no. A2022001). Male BALB/c mice (25 ± 2 g) were purchased from Minhou Wu’s experimental Animal Trading Co., Ltd. (Quanzhou, Fujian, China), acclimatized in an environment with a stable temperature of 22 ± 2 °C and provided with sufficient standard rodent water and chow for at least two weeks. Then, three experimental groups were set up as follows (*n* = 8): the control group (treated with the same amount of sterile normal saline), the FL group (gavaged with 125 mg/kg b. w. of SGF−2), and the FH group (gavaged with 250 mg/kg b. w. of SGF−2). The mice were gavaged with SGF−2 dissolved in physiological saline for 28 consecutive days. Then, the mice were killed using the cervical dislocation method on the last day, and each group of mice was dissected and weighed for body weight and immune-related organ weight (spleen and thymus). The thymus and spleen indexes were calculated by the following formula: thymus/spleen index (mg/g) = weight of thymus (spleen)/body weight.

### 3.5. Subpopulation Analysis of Splenocytes

After removing the red blood cells using a lysis buffer, flow cytometry analysis was used to detect the subsets of T lymphocytes, Tregs, and DCs from the mice splenocytes. The T lymphocytes were analyzed with CD3-APC, CD8-FITC, and CD4-PerCP-Cy5.5. The DCs were stained with CD11c-APC and MHC-II-FITC. The Tregs analysis of splenocytes was conducted with CD4-PerCP-Cy5.5 and Foxp3^+^.

### 3.6. Detection of Gut Microbiota

To detect the composition of the gut bacterial community, 16S ribosome RNA gene sequencing was used. The intestinal contents of the three groups of mice were collected and rapidly frozen in liquid nitrogen before storage at −80 °C. We then commissioned Shanghai Tiny Gene Bio-Tech Co., Ltd. (Shanghai, China) to detect and amplify the V3–V4 regions of 16S rRNA using the general primer pair. Sequences with 97% clustering similarity were assigned to the same operation taxonomic unit (OUT). Subsequently, α-diversity analysis, species taxonomy analysis, and different species screening were performed.

### 3.7. SGF−2 Fermentation In Vitro

In vitro fermentation was performed using mice fecal microbiota in an anaerobic culture system [38]. Fresh feces of mice were collected and mixed with sterilized normal saline (*w*/*v*, 20%). The mixed fecal solution was then filtered with gauze, and the insoluble particles were removed by centrifugation (5 min, 500 rpm). The remaining supernatants were collected for in vitro SGF−2 fermentation. Sterilized Erlenmeyer flasks were filled with 9 mL of sterilized basal medium (pH 7.6) and rinsed with oxygen-free N_2_ to maintain anaerobic conditions. The basal medium included yeast extract (2.0 g/L), peptone (2.0 g/L), hemin (0.02 g/L), _L_-cysteine (0.5 g/L), bile salts (0.5 g/L), NaHCH_3_ (2.0 g/L), NaCl (0.1 g/L), KH_2_PO_4_ (0.04 g/L), K_2_HPO_4_ (0.04 g/L), CaCl_2_·6H_2_O (0.01 g/L), MgSO_4_·7H_2_O (0.01 g/L), vitamin K1 (0.01 g/L), resazurin (0.01 g/L), and Tween-80 (2.0 mL/L). The pH of the fermentation medium was adjusted to 7.6 and sterilized at 121 °C for 20 min. The fecal slurry (1.0 mL) and 50 mg of SGF−2 were inoculated into the appropriate fermentation flask. In addition, fermentation flasks with or without LPS were included as the experimental group and positive control, respectively. After fermentation (0, 12, 24, and 36 h), samples were collected and centrifuged to remove the precipitation (15 min, 8000 rpm). The remaining supernatants were then passed through a 0.22 μm filter membrane and stored at −20 °C before use.

### 3.8. Cell Culture

A human intestinal epithelial Caco-2 cell line and murine macrophage RAW264.7, obtained from the Chinese Academy of Sciences Cell Bank (Shanghai, China), were suspended in DMEM supplemented with 10% (*v*/*v*) FBS, 100 μg/ml of streptomycin, and 100 units/ml of penicillin in a humidified atmosphere of 5% CO_2_ at 37 °C.

### 3.9. Caco-2/RAW264.7 Co-Culture Model

The Caco-2/RAW264.7 co-culture systems were performed on 24-well Transwell plates (Corning, Cambridge, MA, USA). The Caco-2 cells were inoculated at 1 × 10^5^ cells/well in the apical chamber of the Transwell, and the culture medium was changed every other day. When the trans-epithelial resistance (TEER) value of the Caco−2 cells was above 300 Ω·cm^2^, 1 × 10^5^ RAW264.7 cells were inoculated in the basal chamber of the Transwell. After co-culture for 24 h, the LPS or SGF−2 fermentation supernatant (supernatants were diluted with culture medium at the ratio of 1:1000) was added to the apical chamber of the Transwell. In the control group, the fermentation supernatant without SGF−2 was added to the apical chamber of the Transwell. After co-culture for another 24 h, the culture medium or RAW264.7 macrophages in the basal chamber were collected for subsequent experiments.

### 3.10. Cell Viability Measurements by CCK-8

After treatment, a cell counting kit (CCK-8) was used to measure the cell viability of RAW264.7 in the basal chamber. Briefly, an amount of CCK-8 solution (10 μL per 100 μL) was added to each well and incubated for another 2 h. Finally, the absorbance at 450 nm was read using a TECAN microplate reader.

### 3.11. NO and Cytokine Production Assay

After the treatment, the corresponding ELISA kits were used to quantify the levels of cytokines (TNF-α, IL-10, and IL-6) in the basal culture medium. Briefly, 50 μL of sample or standard was added to the appropriate wells, and then 50 μL of the antibody cocktail was added to each well. The plate was incubated on a plate shaker for 1 h at room temperature. Then, each well was washed with wash buffer three times; 100 μL of TMB development solution was added to incubate for 10 min; and then 100 μL stop solution was added to each well. Finally, the absorbance at 450 nm was read. The cytokine levels were calculated based on the standard curve.

The Griess method was used to determine the NO production in the basal culture medium. Briefly, 50 μL of sample or standard was added to the appropriate wells, and then 50 μL of Griess reagent I and 50 μL of Griess reagent II were sequentially added to each well. Finally, the absorbance at 540 nm was read. The concentration of NO in the sample was calculated based on the standard curve.

### 3.12. Phagocytic Activity Assay

The phagocytosis of RAW264.7 macrophages was measured by the neutral red uptake [39]. After the treatment, the basal culture medium was removed, and neutral red (0.075%, 100 μL/well) was added and incubated for another 4 h. Then, the cells were washed three times with PBS and added to a 50% ethanol solution (*v*/*v*) containing 1% acetic acid (*v*/*v*) for 10 min. Finally, a TECAN microplate reader was used to assess the optical density at 540 nm.

### 3.13. Statistical Analysis

GraphPad Prism 8.01 was used for mapping and data analysis. One-way ANOVA and Tukey tests were used to analyze the significant differences. *p* < 0.05 (two-sided) was regarded as a statistically significant difference. All the experimental data were presented by the mean ± SD.

## 4. Conclusions

In general, two fractions SGF−1 and SGF−2 were obtained from the SGFs: SGF−1 was a polysaccharide with a 113 kDa average molecular weight, 74.72% total sugar content, and 5.00% sulfate content; SGF−2 was a polysaccharide with a 258.07 kDa average molecular weight, 55.79% of total sugar content, and 31.81% of sulfate content. The high-yield SGF−2 was further investigated for its in vivo and in vitro immunomodulatory activities. The in vivo experiments indicated that SGF−2 can increase the spleen and thymus indices, the subsets of T lymphocytes, and Tregs in mice. The 16S high-throughput sequencing of mice intestinal contents showed that oral administration of SGF−2 did not significantly change the diversity of gut microbiota, but did change the composition of intestinal microorganisms, especially the proportion of probiotics. Moreover, SGF−2 fermented by feces improved the phagocytic ability and promoted the NO and cytokine production of RAW264.7 macrophages in the co-culture system. Overall, the present study verifies the effects of fucoidan from *S. graminifolium* on the immune modulatory effects and gut microbiota, which could promote the development of *S. graminifolium* pharmaceuticals.

## Figures and Tables

**Table 1:** Chemical composition of SGF−1 and SGF−2 isolated from *S. graminifolium*.

Sample	Sugar Content (%)	Proteins (%)	Sulfate Content (%)	Mw/kDa	Monosaccharide Composition (%)					
					Fuc	Gal	Glc	Xyl	Man	Glc-UA
SGF−1	74.72 ± 0.37	0.92 ± 0.47	5.00 ± 0.15	112.96	40.54	10.50	2.30	10.93	22.80	12.93
SGF−2	55.79 ± 0.26	0.69 ± 0.23	31.81 ± 0.99	258.07	35.57	21.52	0.28	7.21	21.77	13.65

**Table 2:** The effect of SGF−2 treatments on immune organ indices and body weight.

Group	Initial Weight (g)	Final Weight (g)	Weight Gain Percentage (%)	Spleen Index (mg/g)	Thymus Index (mg/g)
Con	26.80 ± 0.79	34.82 ± 0.68	29.93%	4.30 ± 0.98	1.63 ± 0.62
FL	26.72 ± 0.93	34.93 ± 0.63	30.72%	4.67 ± 0.39	1.68 ± 0.59
FH	27.13 ± 0.56	35.24 ± 0.55	29.90%	5.34 ± 0.58 (*)	2.07 ± 0.75 (*)

Con, mice gavaged at the same volume of sterile physiological saline. FL, mice gavaged at low dose SGF−2 (125 mg/kg body weight). FH, mice gavaged at high dose SGF−2 (250 mg/kg body weight). Data are expressed as mean ± SD (*n* = 8). * *p* < 0.05 vs. Con group.

## Data Availability

Data are contained within the article and supplementary materials.

## Supplementary Materials

The supporting information can be downloaded at: https://www.mdpi.com/article/10.3390/molecules28237794/s1. Ion chromatogram of santard samples, SGF−1 and SGF−2 (Figure S1–S3); Molecular weight test results of SGF−1 and SGF−2 (Table S1); RMS conformation plot of SGF−1 (Figure S4); Absolute molecular weight analysis plot of SGF−1 (Figure S5); RMA conformation plot of SGF−2 (Figure S6); Absolute molecular weight analysis plot of SGF−1 (Figure S7) are included in the supporting information.

## References

[1] Huang W., Tan H., Nie S. (2022). Beneficial effects of seaweed-derived dietary fiber: Highlights of the sulfated polysaccharides. Food Chem..

[2] Koh A., De Vadder F., Kovatcheva-Datchary P., Bäckhed F. (2016). From Dietary Fiber to Host Physiology: Short-Chain Fatty Acids as Key Bacterial Metabolites. Cell.

[3] Tagliapietra B.L., Clerici M.T.P.S. (2023). Brown algae and their multiple applications as functional ingredient in food production. Food Res. Int..

[4] Ale M.T., Mikkelsen J.D., Meyer A.S. (2011). Important determinants for fucoidan bioactivity: A critical review of structure-function relations and extraction methods for fucose-containing sulfated polysaccharides from brown seaweeds. Mar. Drugs.

[5] Zhang C.-Y., Kong T., Wu W.-H., Lan M.-B. (2013). The Protection of Polysaccharide from the Brown Seaweed Sargassum graminifolium against Ethylene Glycol-Induced Mitochondrial Damage. Mar. Drugs.

[6] Zhang C.Y., Wu W.H., Wang J., Lan M.B. (2012). Antioxidant properties of polysaccharide from the brown seaweed *Sargassum graminifolium* (Turn.), and its effects on calcium oxalate crystallization. Mar Drugs.

[7] Huang L., Zeng Q., Zhang Y., Yin Q., Zhu X., Zhang P., Wang C., Liu J. (2022). Effects of fucoidans and alginates from *Sargassum graminifolium* on allergic symptoms and intestinal microbiota in mice with OVA-induced food allergy. Food Funct..

[8] Liu C., Du P., Cheng Y., Guo Y., Hu B., Yao W., Zhu X., Qian H. (2021). Study on fecal fermentation characteristics of aloe polysaccharides in vitro and their predictive modeling. Carbohydr. Polym..

[9] Luo X., Huang Q., Fu X., Kraithong S., Hu Y., Yuan Y., Bao J., Zhang B. (2023). In vitro fecal fermentation characteristics of mutant rice starch depend more on amylose content than crystalline structure. Carbohydr. Polym..

[10] Bogdanov I.V., Finkina E.I., Melnikova D.N., Ziganshin R.H., Ovchinnikova T.V. (2021). Investigation of Sensitization Potential of the Soybean Allergen Gly m 4 by Using Caco-2/Immune Cells Co-Culture Model. Nutrients.

[11] Zhu M., Huang R., Wen P., Song Y., He B., Tan J., Hao H., Wang H. (2021). Structural characterization and immunological activity of pectin polysaccharide from kiwano (*Cucumis metuliferus*) peels. Carbohydr. Polym..

[12] Nicholson J.K., Holmes E., Kinross J., Burcelin R., Gibson G., Jia W., Pettersson S. (2012). Host-gut microbiota metabolic interactions. Science.

[13] Ishiguro E., Haskey N., Campbell K., Ishiguro E., Haskey N., Campbell K. (2018). Chapter 4—Gut Microbiota in Health and Disease. Gut Microbiota.

[14] Mahowald M.A., Rey F.E., Seedorf H., Turnbaugh P.J., Fulton R.S., Wollam A., Shah N., Wang C., Magrini V., Wilson R.K. (2009). Characterizing a model human gut microbiota composed of members of its two dominant bacterial phyla. Proc. Natl. Acad. Sci. USA.

[15] Turnbaugh P.J., Ridaura V.K., Faith J.J., Rey F.E., Knight R., Gordon J.I. (2009). The effect of diet on the human gut microbiome: A metagenomic analysis in humanized gnotobiotic mice. Sci. Transl. Med..

[16] Fernández J., Redondo-Blanco S., Gutiérrez-del-Río I., Miguélez E.M., Villar C.J., Lombó F. (2016). Colon microbiota fermentation of dietary prebiotics towards short-chain fatty acids and their roles as anti-inflammatory and antitumour agents: A review. J. Funct. Foods.

[17] Smith P.M., Howitt M.R., Panikov N., Michaud M., Gallini C.A., Bohlooly Y.M., Glickman J.N., Garrett W.S. (2013). The microbial metabolites, short-chain fatty acids, regulate colonic Treg cell homeostasis. Science.

[18] Sakaguchi S., Yamaguchi T., Nomura T., Ono M. (2008). Regulatory T cells and immune tolerance. Cell.

[19] Park J., Kim M., Kang S.G., Jannasch A.H., Cooper B., Patterson J., Kim C.H. (2015). Short-chain fatty acids induce both effector and regulatory T cells by suppression of histone deacetylases and regulation of the mTOR–S6K pathway. Mucosal Immunol..

[20] Belzer C., de Vos W.M. (2012). Microbes inside--from diversity to function: The case of *Akkermansia*. ISME J..

[21] Cani P.D., de Vos W.M. (2017). Next-Generation Beneficial Microbes: The Case of *Akkermansia muciniphila*. Front. Microbiol..

[22] Shin N.-R., Lee J.-C., Lee H.-Y., Kim M.-S., Whon T.W., Lee M.-S., Bae J.-W. (2014). An increase in the *Akkermansia* spp. population induced by metformin treatment improves glucose homeostasis in diet-induced obese mice. Gut.

[23] Li X., Jiao L.-L., Zhang X., Tian W.-M., Chen S., Zhang L.-P. (2008). Anti-tumor and immunomodulating activities of proteoglycans from mycelium of Phellinus nigricans and culture medium. Int. Immunopharmacol..

[24] Jin J.-O., Zhang W., Du J.-Y., Wong K.-W., Oda T., Jiyu G. (2014). Fucoidan Can Function as an Adjuvant In Vivo to Enhance Dendritic Cell Maturation and Function and Promote Antigen-Specific T Cell Immune Responses. PLoS ONE.

[25] Hayashi K., Nakano T., Hashimoto M., Kanekiyo K., Hayashi T. (2008). Defensive effects of a fucoidan from brown alga *Undaria pinnatifida* against herpes simplex virus infection. Int. Immunopharmacol..

[26] Tan J., McKenzie C., Vuillermin P.J., Goverse G., Vinuesa C.G., Mebius R.E., Macia L., Mackay C.R. (2016). Dietary Fiber and Bacterial SCFA Enhance Oral Tolerance and Protect against Food Allergy through Diverse Cellular Pathways. Cell Rep..

[27] Green S.J., Mellouk S., Hoffman S.L., Meltzer M.S., Nacy C.A. (1990). Cellular mechanisms of nonspecific immunity to intracellular infection: Cytokine-induced synthesis of toxic nitrogen oxides from _L_-arginine by macrophages and hepatocytes. Immunol. Lett..

[28] Tabarsa M., Dabaghian E.H., You S., Yelithao K., Cao R., Rezaei M., Alboofetileh M., Bita S. (2020). The activation of NF-κB and MAPKs signaling pathways of RAW264.7 murine macrophages and natural killer cells by fucoidan from Nizamuddinia zanardinii. Int. J. Biol. Macromol..

[29] Hwang P.-A., Lin H.-T., Lin H.-Y., Lo S.-K. (2019). Dietary Supplementation with Low-Molecular-Weight Fucoidan Enhances Innate and Adaptive Immune Responses and Protects against *Mycoplasma pneumoniae* Antigen Stimulation. Mar. Drugs.

[30] Mao Y.-H., Song A.-X., Li L.-Q., Yang Y., Yao Z.-P., Wu J.-Y. (2020). A high-molecular weight exopolysaccharide from the Cs-HK1 fungus: Ultrasonic degradation, characterization and in vitro fecal fermentation. Carbohydr. Polym..

[31] Kasubuchi M., Hasegawa S., Hiramatsu T., Ichimura A., Kimura I. (2015). Dietary gut microbial metabolites, short-chain fatty acids, and host metabolic regulation. Nutrients.

[32] Noval Rivas M. (2016). Regulatory T cells in allergic diseases. J. Allergy Clin. Immunol..

[33] Singh N., Gurav A., Sivaprakasam S., Brady E., Padia R., Shi H., Thangaraju M., Prasad P.D., Manicassamy S., Munn D.H. (2014). Activation of Gpr109a, Receptor for Niacin and the Commensal Metabolite Butyrate, Suppresses Colonic Inflammation and Carcinogenesis. Immunity.

[34] DuBois M., Gilles K.A., Hamilton J.K., Rebers P.A., Smith F. (1956). Colorimetric Method for Determination of Sugars and Related Substances. Anal. Chem..

[35] Borazjani N., Tabarsa M., You S., Rezaei M. (2018). Purification, molecular properties, structural characterization, and immunomodulatory activities of water soluble polysaccharides from Sargassum angustifolium. Int. J. Biol. Macromol..

[36] Li Q., Jiang S., Shi W., Qi X., Song W., Mou J., Yang J. (2020). Structure characterization, antioxidant and immunoregulatory properties of a novel fucoidan from the sea cucumber Stichopus chloronotus. Carbohydr Polym.

[37] Hu T., Huang Q., Wong K., Yang H. (2017). Structure, molecular conformation, and immunomodulatory activity of four polysaccharide fractions from Lignosus rhinocerotis sclerotia. Int. J. Biol. Macromol..

[38] Díez-Municio M., Kolida S., Herrero M., Rastall R.A., Moreno F.J. (2016). In vitro faecal fermentation of novel oligosaccharides enzymatically synthesized using microbial transglycosidases acting on sucrose. J. Funct. Foods.

[39] Liu Q.M., Xu S.S., Li L., Pan T.M., Shi C.L., Liu H., Cao M.J., Su W.J., Liu G.M. (2017). In vitro and in vivo immunomodulatory activity of sulfated polysaccharide from Porphyra haitanensis. Carbohydr. Polym..

