# Supplementary material for: Study on Immunoregulatory Effects of Fucoidan from Sargassum graminifolium In Vivo and Immunoactivation Activity of Its Fecal Fermentation Products Using Co-Culture Model
Source: Molecules. 2023 Nov 27;28(23):7794. doi: 10.3390/molecules28237794 (PMC10707906; doi:10.3390/molecules28237794)
Supplement: Supplementary file 1 [file molecules-28-07794-s001.zip › molecules-2724648-supplementary.pdf]

*molecules*

# Study on Immunoregulatory Effects of Fucoidan from *Sargassum graminifolium* in vivo and Immunoactivation Activity of Its Fecal Fermentation Products Using Co-Culture Model

**Cuifang Wang [1,2,*], Lan Huang [3], Yaolong Huang [1], Xin, Tian [1] and Jieqing Liu [3,*]**

[1] College of Oceanology and Food Science, Quanzhou Normal University, Quanzhou 362000, China; huangyaolong@stumail.qztc.edu.cn (Y.H.); 201005017@stumail.qztc.edu.cn (X.T.)
[2] Fujian Province Key Laboratory for the Development of Bioactive Material from Marine Algae, Quanzhou Normal University, Quanzhou 362000, China
[3] School of Medicine, Huaqiao University, Quanzhou 362021, China; hl287216411@163.com
* Correspondence: wangcuifang@qztc.edu.cn (C.W.); liujieqing@hqu.edu.cn (J.L.)

**Monosaccharide composition results of SGF-1 and SGF-2**

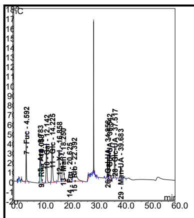


**Figure S1.** Ion chromatogram of standard sample.

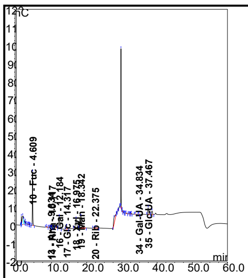


**Figure S2.** Ion chromatogram of SGF-1.

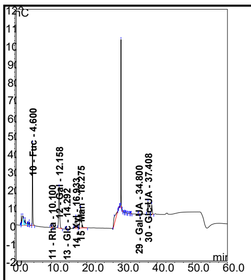


**Figure S3.** Ion chromatogram of SGF-2.

## Molecular weight test results of SGF-1 and SGF-2

**Table S1.** Molecular weight determination of grade SGPF-1and SGPF-2.

| | Mn (kDa) | Mp (kDa) | Mw (kDa) | Mz (kDa) |
|---|---|---|---|---|
| SGF-1 | 23.724 | 250.395 | 112.96 | 454.32 |
| SGF-2 | 138.514 | 255.486 | 258.066 | 606.787 |

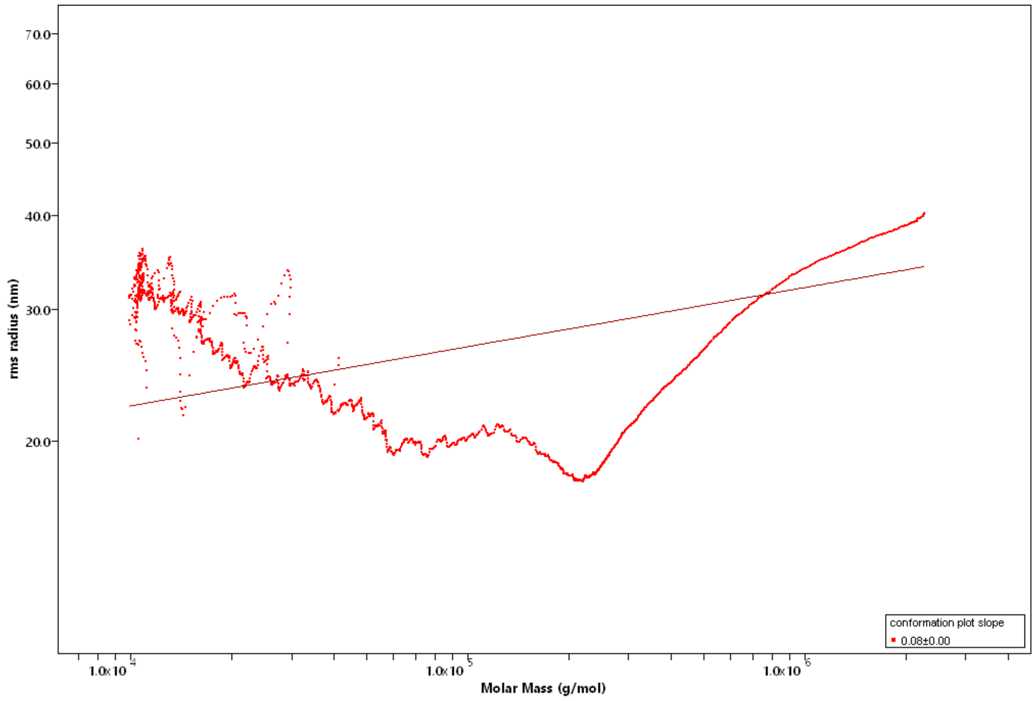


**Figure S4.** RMS conformation plot of SGF-1.

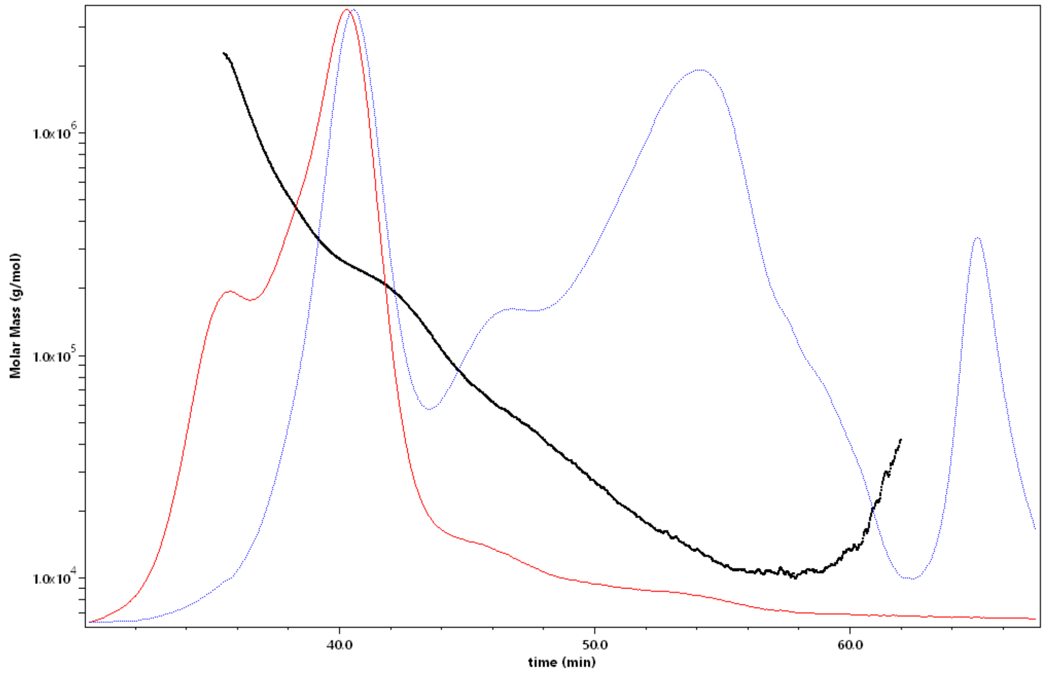


**Figure S5.** Absolute molecular weight Analysis plot of SGF-1.

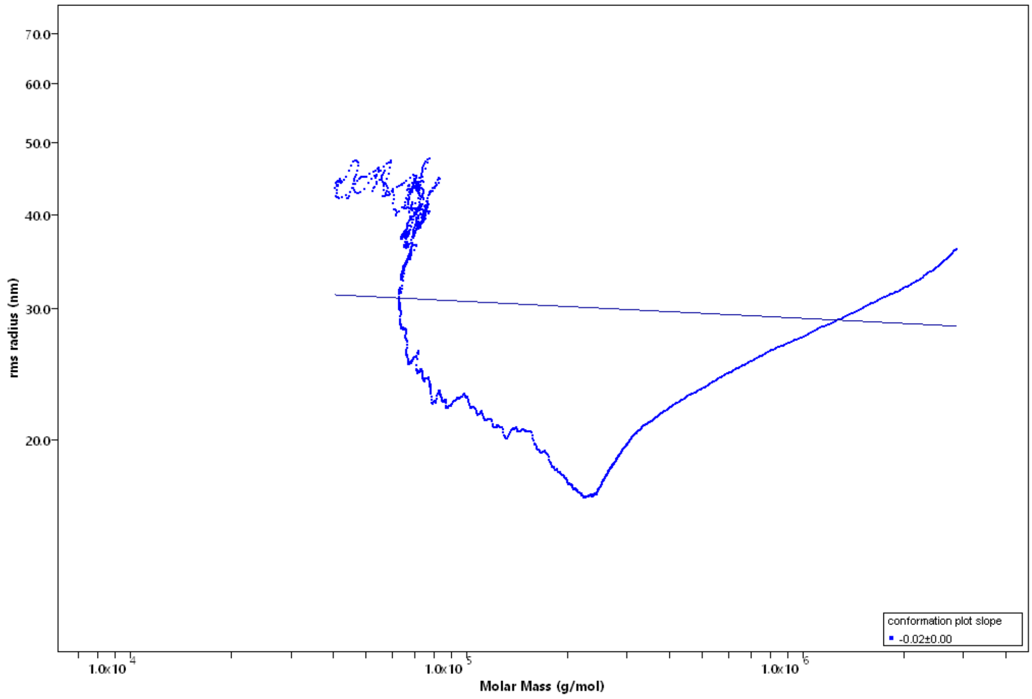


**Figure S6.** RMS conformation plot of SGF-2.

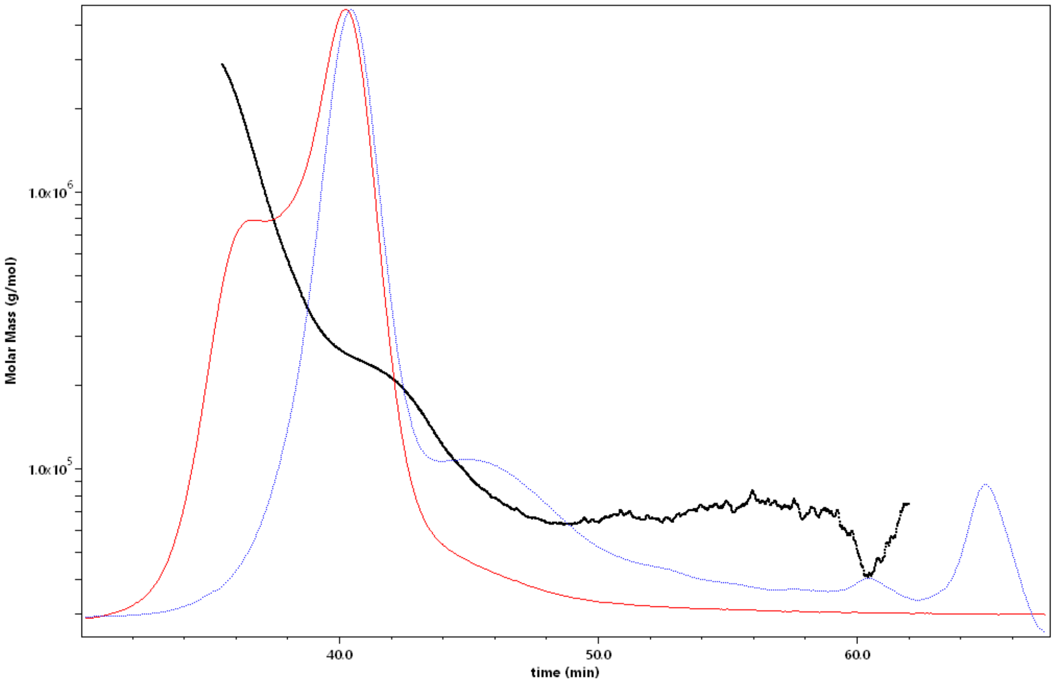


**Figure S7.** Absolute molecular weight Analysis plot of SGF-2.
